# BLISS is a versatile and quantitative method for genome-wide profiling of DNA double-strand breaks

**DOI:** 10.1038/ncomms15058

**Published:** 2017-05-12

**Authors:** Winston X. Yan, Reza Mirzazadeh, Silvano Garnerone, David Scott, Martin W. Schneider, Tomasz Kallas, Joaquin Custodio, Erik Wernersson, Yinqing Li, Linyi Gao, Yana Federova, Bernd Zetsche, Feng Zhang, Magda Bienko, Nicola Crosetto

**Affiliations:** 1 Broad Institute of MIT and Harvard, Cambridge, Massachusetts 02142, USA; 2 Graduate Program in Biophysics, Harvard Medical School, Boston, Massachusetts 02115, USA; 3 Harvard-MIT Division of Health Sciences and Technology, Harvard Medical School, Boston, Massachusetts 02115, USA; 4 Science for Life Laboratory, Division of Translational Medicine and Chemical Biology, Department of Medical Biochemistry and Biophysics, Karolinska Institutet, Stockholm SE-17165, Sweden; 5 McGovern Institute for Brain Research, Massachusetts Institute of Technology, Cambridge, Massachusetts 02139, USA; 6 Department of Brain and Cognitive Sciences, Massachusetts Institute of Technology, Cambridge, Massachusetts 02139, USA; 7 Department of Biological Engineering, Massachusetts Institute of Technology, Cambridge, Massachusetts 02139, USA

## Abstract

Precisely measuring the location and frequency of DNA double-strand breaks (DSBs) along the genome is instrumental to understanding genomic fragility, but current methods are limited in versatility, sensitivity or practicality. Here we present Breaks Labeling *In Situ* and Sequencing (BLISS), featuring the following: (1) direct labelling of DSBs in fixed cells or tissue sections on a solid surface; (2) low-input requirement by linear amplification of tagged DSBs by *in vitro* transcription; (3) quantification of DSBs through unique molecular identifiers; and (4) easy scalability and multiplexing. We apply BLISS to profile endogenous and exogenous DSBs in low-input samples of cancer cells, embryonic stem cells and liver tissue. We demonstrate the sensitivity of BLISS by assessing the genome-wide off-target activity of two CRISPR-associated RNA-guided endonucleases, Cas9 and Cpf1, observing that Cpf1 has higher specificity than Cas9. Our results establish BLISS as a versatile, sensitive and efficient method for genome-wide DSB mapping in many applications.

## Abstract (web summary)

Double-strand breaks are a major DNA lesion that can occur by endogenous and exogenous processes. Here the authors present BLISS—Breaks Labelling *In Situ* and Sequencing—to map breaks across the genome.

DNA double-strand breaks (DSBs) are major DNA lesions that form in a variety of physiological conditions—such as transcription^1^,^2^, meiosis^3^ and VDJ recombination^4^—as well as a consequence of exposure to DNA-damaging agents and replication stress^5^. DSBs can also be induced in a controlled manner at specific sites in the genome using programmable nucleases, such as the CRISPR (clustered regularly interspaced short palindromic repeats)-associated RNA-guided endonucleases, Cas9 and Cpf1, which have greatly advanced genome editing. However, the potentially mutagenic off-target DNA cleavage activity of these nucleases represents an issue of major concern that needs to be thoroughly assessed before these enzymes can be safely used in the clinical setting^6^. Thus, developing methods that can accurately map the genome-wide location of endogenous as well as exogenous DSBs in different systems and conditions is not only essential to advance our understanding of DSB biology, but is also critical for successful translation of programmable nucleases from research tools into clinical applications.

In the past few years, several methods based on next-generation sequencing (NGS) have been developed to assess DSBs at genomic scale, including chromatin immunoprecipitation sequencing^7^,^8^, direct in situ breaks labeling, enrichment on streptavidin and next-generation sequencing (BLESS)^9^,^10^,^11^, genome-wide, unbiased identification of DSBs enabled by sequencing (GUIDE-seq)^12^, *in vitro* Cas9-digested whole-genome sequencing (Digenome-seq)^13^, integrase-defective lentiviral vector (IDLV)-mediated DNA break capture^14^, high-throughput, genome-wide, translocation sequencing^15^ and more recently End-Seq^16^ and DSBCapture^17^. Although all of these methods represent important complementary tools to detect DSBs genome wide (Supplementary Table 1), they also have important drawbacks. For example, chromatin immunoprecipitation sequencing of DSB-sensing or repair proteins such as p53-binding protein 1 or the phosphorylated variant histone H2A.X (γH2A.X) does not label DSBs directly and is unable to identify DNA breakpoints with single-nucleotide resolution. GUIDEseq, IDLV-mediated DNA break capture and high-throughput, genome-wide, translocation sequencing detect DSBs by quantifying the products of non-homologous end-joining repair, potentially missing DSBs that are repaired through other pathways. Furthermore, *in vivo* delivery of exogenous oligonucleotides in GUIDEseq or viral cassettes in IDLV-mediated DNA break capture for evaluating DSBs in primary cells and intact tissues may be challenging. DSBs induced by programmable nucleases, such as CRISPR-associated RNA-guided Cas9 and Cpf1, can be evaluated *in vitro* using Digenome-seq, but this approach may not be representative of relevant nuclease concentrations and of cellular properties, such as chromatin environment and nuclear architecture, which might influence the frequency of DNA breaking and repair. Lastly, BLESS and the related methods End-Seq^16^ and DSBCapture^17^ require substantial amounts of input material (typically, in the order of millions of cells), are labour-intensive and are semi-quantitative due to lack of appropriate controls for PCR amplification biases, limiting their applications and scalability. Here we describe a method for breaks labeling *in situ* and sequencing (BLISS) that compared with other DSB mapping methods is more versatile, sensitive and quantitative. We demonstrate the broad applicability of BLISS for genome-wide detection of both endogenous and exogenous DSBs in low-input samples of cells and tissues, as well as for genome-wide profiling of on- and off-target DSBs introduced by Cas9 and Cpf1 nucleases.

## Results

### BLISS implementation and validation

**Figure 1:**
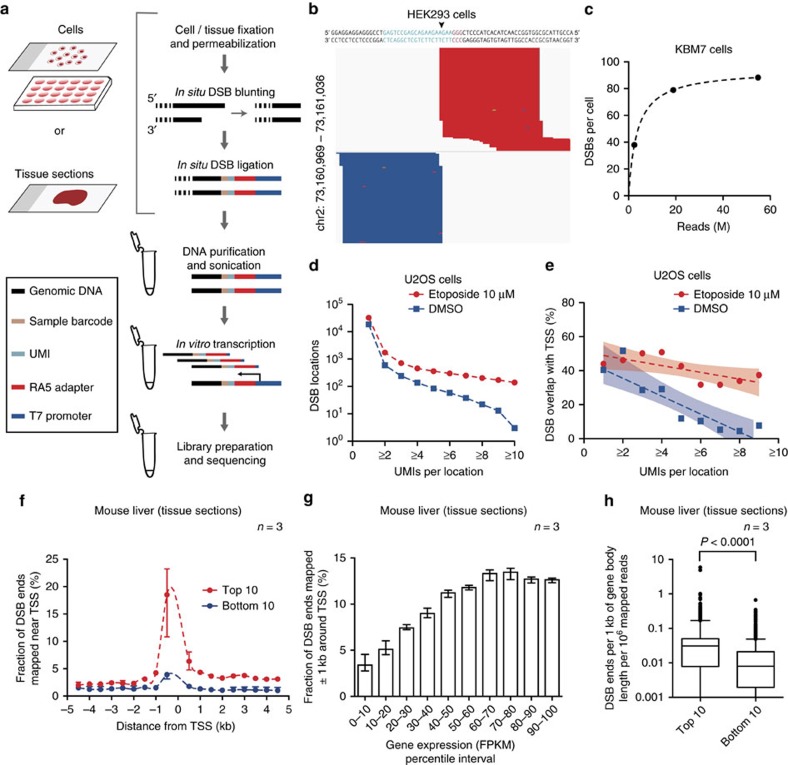
Quantitative detection of natural and etoposide-induced DSBs. (**a**) Schematic of BLISS. The workflow starts by either fixing cells onto a microscope slide or in a multi-well plate, or by immobilizing already fixed tissue sections onto a slide. DSB ends are then *in situ* blunted and tagged with dsDNA adapters containing components described in the boxed legend and in Supplementary Data 1. Tagged DSB ends are linearly amplified using *in vitro* transcription and the resulting RNA is used for Illumina library preparation and sequencing. (**b**) BLISS reads aligned to an SpCas9 on-target cut site (arrowhead) in the *EMX1* gene. Light blue, guide sequence. Orange, PAM sequence. Dark blue, reads mapped to the minus strand. Red, reads mapped to the plus strand. (**c**) Estimated number of DSBs per cell in three replicates sequenced at increasing sequencing depth. Dashed line, hyperbolic interpolation. (**d**) Number of DSB locations in etoposide-treated versus control U2OS cells by filtering on the minimum number of UMIs per DSB location. (**e**) Fraction of DSB locations mapped around the transcription start sites (TSS) in control versus etoposide-treated U2OS cells as a function of the minimum number of UMIs per DSB location. Dashed lines, linear interpolation. Colour shades, 95% confidence intervals. (**f**) For BLISS on mouse liver, mapping of sequenced DSB ends found in the top 10% (red) and bottom 10% (blue) of expressed genes in the mouse liver. *n*, number of biological replicates. Dots, mean value. Whiskers, min-max range. Dashed lines, spline interpolation. (**g**) Percentage of sequenced DSB ends mapped in a ±1 kb interval around the TSS for each inter-decile interval of gene expression in mouse liver. FPKM, fragments per kilobase of transcript per million mapped reads. *n*, number of biological replicates. Bars, mean value. Whiskers, min–max range. (**h**) Number of sequenced DSB ends mapped per kilobase inside the gene body of the top 10% and bottom 10% expressed genes in mouse liver. *n*, number of biological replicates. Whiskers, 2.5–97.5 percentile range. *P*, Mann–Whitney test.

A detailed workflow of the BLISS method is depicted in Fig. 1a and a step-by-step protocol can be found in Protocol Exchange^18^. Briefly, the procedure starts by attaching cells or tissue sections fixed with formaldehyde onto a microscope slide or coverglass, which enables all the subsequent *in situ* reactions to be performed without centrifugations, thus minimizing the risk of introducing artificial DNA breaks and sample loss. DSBs are *in situ* blunted and then ligated with a double-stranded DNA oligonucleotide adapter containing the T7 promoter sequence, the RA5 Illumina sequencing adapter, a random stretch of 8–12 nucleotides (nt) that serves as unique molecular identifier (UMI)^19^ and a sample barcode suitable for multiplexing (Supplementary Fig. 1a and Supplementary Data 1). Following genomic DNA (gDNA) extraction, the sequence immediately downstream to the tagged DSBs is linearly amplified via T7-mediated *in vitro* transcription, which has been shown to introduce fewer biases compared with exponential amplification by PCR when amplifying complementary DNA from low-input samples including single cells^20^,^21^.

Overall, application of BLISS to various sample types and preparations as described below yielded high-quality sequencing libraries with a balanced UMI and strand composition (Supplementary Fig. 1b–d). For most of the samples, we performed single-end sequencing (Supplementary Data 2). We developed a pre-processing pipeline that, by using the information contained in the UMIs, filters out PCR duplicates without the need for paired-end sequencing and counts DSB events that have occurred at the same genomic location in multiple cells (Supplementary Fig. 1e–g and Methods).

We first tested whether BLISS can faithfully detect DSBs occurring at defined locations in the genome, even in low-input samples of few thousand cells. We transfected HEK293 cells with *Streptococcus pyogenes* Cas9 (SpCas9) and a single-guide RNA (sgRNA) targeting the *EMX1* gene. BLISS was able to precisely localize and quantify both DSB ends generated by SpCas9 at the correct on-target location (Fig. 1b). Furthermore, in low-input samples of KBM7 cells, BLISS precisely identified telomeric ends, which mimic DSB ends, and was able to reproduce the frequency distribution of the 5′ recessed telomeric ends previously identified in a much larger number of cells using BLESS^9^ (Supplementary Fig. 2a).

We then assessed the accuracy and quantitative power of BLISS by sequencing at increasing depth three libraries obtained from low-input samples of KBM7 cells (Supplementary Data 2). By performing rarefaction analysis on the number of unique DSBs labelled by UMIs that were detected at increasing sequencing depths, we estimated that BLISS was able to detect 80–100 DSBs per cell (Fig. 1c and Methods). This estimate was within the same range of the number of γH2A.X foci quantified by microscopy in the same cell line (85.7±60.6 foci per cell, mean±s.d., Supplementary Fig. 2b,c), suggesting that most of the DSBs detected by BLISS represent true biological events rather than background noise.

To further assess the quantitative ability of BLISS, we used UMIs to count DSBs induced by the topoisomerase inhibitor, etoposide. In two biological replicates of U2OS cells treated with etoposide, the number of unique DSB ends detected by BLISS increased in a dose-dependent manner, consistent with γH2A.X measurements (Supplementary Fig. 3a–c). The treatment resulted in DSB accumulation at recurrent genomic locations in multiple cells, which could be distinguished thanks to the fact that multiple DSB ends mapping to the same location were labelled by distinct UMIs (Fig. 1d and Supplementary Fig. 3d,e). These recurrent locations were significantly enriched in the neighborhood of transcriptional start sites (TSS), confirming prior findings by BLESS that etoposide has prominent effects around TSS^22^ (Fig. 1e and Supplementary Fig. 3f).

### Profiling of endogenous DSBs in primary cells and tissue

The ability to obtain genome-wide DSB maps from primary cells and tissue samples would greatly help studies of DNA damage and repair processes in animal models and clinical samples. With this goal in mind, we performed proof-of-principle experiments using either tissue sections or purified nuclei derived from mouse liver biopsies (Supplementary Fig. 4a,b). In line with recent findings in different cell types^1^,^2^,^17^, DSBs were strongly enriched in the neighbourhood of the TSS, as well as along the gene body of highly expressed genes (Fig. 1f–h and Supplementary Fig. 4c–e). Gene Ontology analysis of those genes that were reproducibly identified as carrying the highest DSB levels in three biological replicates revealed a significant enrichment in functional terms related to liver-specific metabolic processes, indicating that BLISS is able to capture endogenous DSBs related to tissue-specific processes (Supplementary Fig. 4f and Supplementary Data 3 and 4). A similar enrichment of DSBs in the neighbourhood of the TSS and along the gene body of highly expressed genes was also recapitulated in low-input samples of primary mouse embryonic stem cells (mESCs) (Supplementary Fig. 4g–i), confirming that BLISS is a highly versatile method that can be applied to study endogenous DSBs in various cell and tissue samples. Furthermore, we assessed chromatin accessibility in liver tissue sections adjacent to those processed by BLISS, by applying a modified BLISS protocol in which artificial DNA breaks are first introduced *in situ* by the HindIII restriction endonuclease (Supplementary Fig. 5a,b, Methods and Protocol Exchange^18^. This revealed that, although endogenous DSBs mapped by BLISS were enriched in the open chromatin regions characterized by a high frequency of HindIII cuts, in analogy to previous findings^16^,^17^, many genomic regions with similar chromatin accessibility had very different DSB levels and *vice vers*a (Supplementary Fig. 5c).

### Profiling of Cas9 and Cpf1 specificity

We next aimed to assess the sensitivity of BLISS by characterizing the DSBs induced by Cas9 and Cpf1. Evaluating Cas9 and Cpf1 on- and off-targets is a valuable way of assessing BLISS sensitivity, because the nuclease-induced cleavage sites (1) are sparse enough so as to not saturate BLISS; (2) are relatively well-defined by both location of cut sites found by other assays^12^,^13^ and the observation that off-targets generally have homology to the on-target guide^9^,^10^,^11^,^12^,^13^; and (3) occur over a wide dynamic range of DSB frequencies to allow quantification of the detection sensitivity. Meanwhile, BLISS is a versatile and minimally disruptive technique for studying the specificity of CRISPR nucleases, as by labelling DSBs post fixation it requires no additional perturbations to the cell beyond delivery of the nuclease and RNA guide. Hence, we developed a workflow to screen the off-target activity of Cas9 or Cpf1 endonucleases using BLISS (Cas9-BLISS and Cpf1-BLISS) in parallel with existing genome-editing protocols (Supplementary Fig. 6a). Aside from culturing cells for BLISS on poly-D-lysine-coated plates and fixation 24 h post transfection, no additional modifications of delivery reagents or workflows were necessary, allowing BLISS to capture a snapshot of the CRISPR nuclease activity in cells with minimal bias.

**Figure 2:**
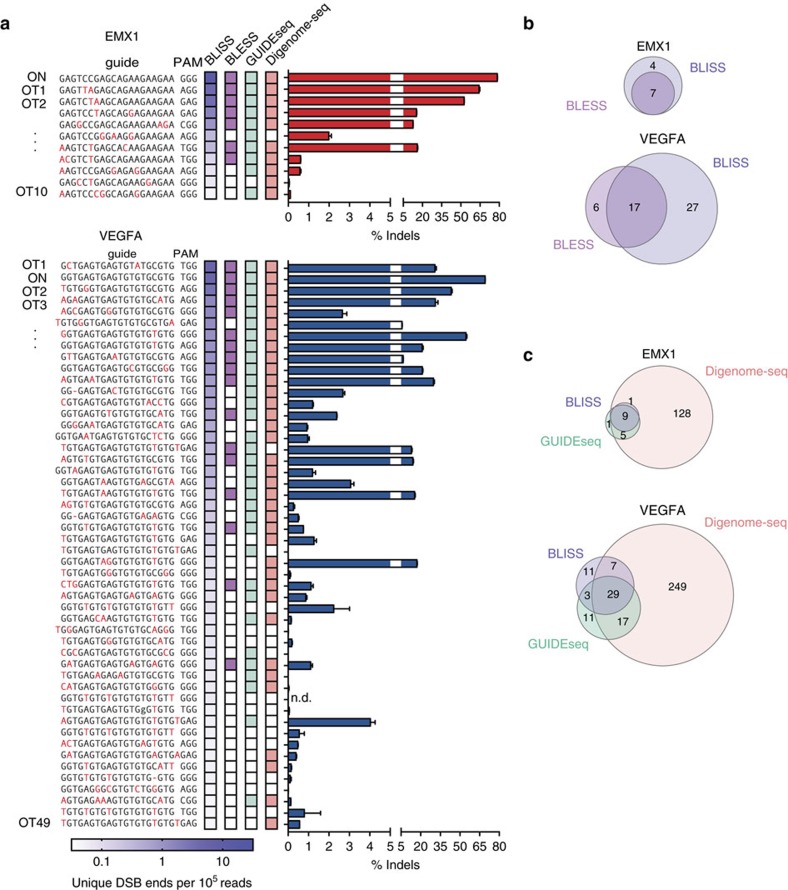
Evaluation of BLISS sensitivity through genome-wide quantification of SpCas9 on- and off-target DSBs. (**a**) On- and off-target sites identified by BLISS, BLESS, GUIDEseq and Digenome-seq. BLISS targets were ranked in descending order based on the number of unique DSB ends aligned to the target per 10^5^ unique BLISS reads. Coloured boxes in the BLESS, GUIDEseq and Digenome-seq columns indicate when the BLISS target was previously found by either of these methods. Individual sites were validated by targeted deep sequencing and the percentage of reads containing an insertion or deletion (indel) is shown. (*n=3*, error bars show s.e.m.). ON, on-target. OT, off-target. (**b**) Overlap between on- and off-target sites identified by BLISS versus BLESS. (**c**) Overlap between on- and off-target sites identified by BLISS versus GUIDEseq and Digenome-seq.

To benchmark the sensitivity of Cas9-BLISS against existing genome-wide specificity methods such as BLESS, GUIDEseq and Digenome-seq, we transfected HEK293 cells with SpCas9 and two sgRNAs targeting the *EMX1* and *VEGFA* genes, both of which have been characterized using all three methods^11^,^12^,^14^,^15^. This set of known off-targets allowed us to further optimize Cas9-BLISS through direct comparison of different DSB labelling strategies, showing that *in situ* A-tailing before adapter ligation increases the sensitivity of DSB detection when directly compared with the original blunt end ligation chemistry (Supplementary Fig. 6b–e). Furthermore, to achieve greater sensitivity we refined the computational pipeline that we previously established for identifying *bona fide* Cas9 DSBs for the analysis of Cas9-BLESS data^10^ (Methods). In addition to the expected on-target DSB sites, BLISS detected numerous off-target sites that were successfully validated by targeted NGS, including many sites previously identified by BLESS, GUIDEseq or Digenome-seq (Fig. 2a and Supplementary Data 5). BLISS also uncovered numerous new off-target sites that were not found in BLESS, even when the refined computational pipeline was re-applied to published BLESS data on the same targets^11^ (Fig. 2b). Side-by-side comparison of BLISS with Digenome-seq and GUIDEseq revealed that although all the three methods generally agree on the top off-targets identified, they differ in the number of weaker off-target sites, particularly in the case of *VEGFA* (Fig. 2c).

We next applied BLISS to characterize the DNA-targeting specificity of Cpf1 (Cpf1-BLISS). Cpf1 is a two-component RNA-programmable DNA nuclease with several unique properties that may broaden the applications of genome engineering: (1) it employs a short CRISPR RNA without an additional trans-activating CRISPR RNA; (2) it utilizes a T-rich protospacer-adjacent motif (PAM) located 5′ to the target sequence; and (3) it generates a staggered cut with a 5′-overhang^23^. We selected six Cpf1 targets across four different genes for genome-wide off-target evaluation using BLISS and targeted NGS. Four targets have NGG PAMs on the 3′-end to enable a simultaneous comparison between SpCas9 and eSpCas9. We evaluated Cpf1 from *Acidaminococcus* sp. (AsCpf1) and *Lachnospiraceae bacterium* (LbCpf1), both of which have been harnessed for efficient mammalian genome editing^23^. At the dual Cpf1 and Cas9 targeted loci, BLISS revealed differences in the *in vivo* pattern of DSBs induced by these two enzymes. Taking the histogram of all the differences between reads mapping to the opposite sides of the DSBs (Supplementary Fig. 7a) showed that although Cas9 cuts are generally blunt ended or contain 1 nt overhangs, Cpf1 cuts exhibit a wide distribution of overhang lengths depending on the target (Supplementary Fig. 7b). Although *in vitro* cleavage of AsCpf1 and LbCpf1 produces 4–5 nt 5′-overhangs as the predominant cleavage outcome^23^, these results suggest that *in vivo* processing of Cpf1 cut sites generates more heterogeneous DSB patterns.

**Figure 3:**
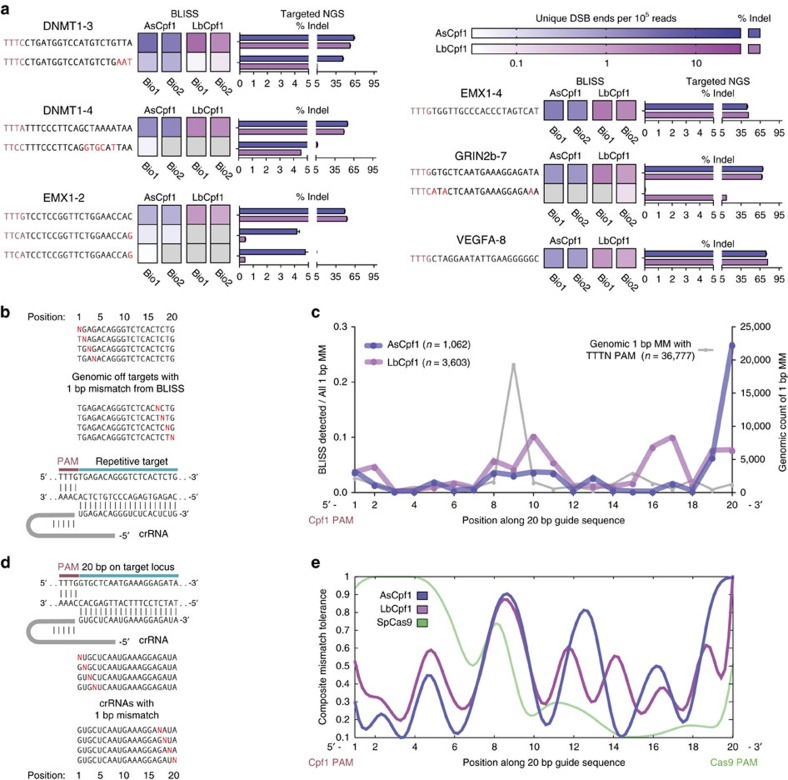
Characterization of AsCpf1 and LbCpf1 specificity. (**a**) Validated on- and off-target sites for AsCpf1 and LbCpf1 for six separate guide targets as measured by Cpf1-BLISS over two independent biological replicates and validated by targeted NGS (*n*=3, error bars show s.e.m.). Grey boxes indicate DSB loci not detected within a biological replicate. (**b**) Evaluating the position-dependent mismatch tolerance of AsCpf1 and LbCpf1 using a repetitive guide with 36,777 predicted genomic loci with single mismatches. (**c**) A map of mismatch tolerance per position generated by dividing at each base the number of off-targets discovered in BLISS versus the possible single mismatched genomic targets for Cpf1. The grey line plotted on the left *y* axis is the count of single mismatched targets in the genome for Cpf1 as predicted by Cas OFFinder^24^. (**d**) Guide designs for investigating the effect of single base pair mismatches in the RNA guide on AsCpf1 and LbCpf1 specificity by measuring the change in their on-target efficiency versus a matched guide. (**e**) Composite mismatch tolerance model for AsCpf1 and LbCpf1 based on saturated single base pair mismatches for two guides. Cas9 data (green) modelled from existing Cas9 single mismatch data^27^.

To identify Cpf1 off-target sites using BLISS, we applied the same computational pipeline as was used for Cas9-BLISS. To maximize sensitivity, we performed targeted NGS on all the off-target sites that were identified in independent BLISS biological replicates from both AsCpf1 and LbCpf1 (Supplementary Fig. 8). Comparing the BLISS results for AsCpf1 or LbCpf1 with SpCas9, we consistently found fewer *bona fide* off-target sites for the two Cpf1 orthologues (Fig. 3a and Supplementary Fig. 8), suggesting that Cpf1 is less tolerant of mismatches than Cas9. For the four targets with shared Cpf1 and Cas9 PAMs, genome modification with SpCas9 yielded a greater range of *bona fide* off-target sites (Supplementary Fig. 9), consistent with prior observations that individual SpCas9 guides can have a wide variation in the number of off-target sites independent of the prevalence of closely matched sites in the genome^12^. As expected, the use of eSpCas9 (ref. 11) reduced the number of off-targets without loss of on-target activity. Lastly, to assess whether BLISS is sensitive enough to detect a large number of Cpf1-induced breaks across a wide dynamic range of cleavage activity, we designed additional guides for Cpf1, targeting repetitive sequences with 278 (*GRIN2b* repetitive guide) and 8,130 (*DNMT1* repetitive guide) perfectly matched on-target sites with a TTTN PAM, as predicted using Cas-OFFinder^24^. A wide range of both on- and off-target loci were detected using Cpf1-BLISS (Supplementary Fig. 10), suggesting that the specificity of Cpf1 determined using BLISS was not an artefact of BLISS, and that Cpf1 can indeed have a high level of specificity for guides not targeting repetitive regions. Altogether, these results corroborate the findings of other recent studies that Cpf1 can be highly specific^25^,^26^.

The Cpf1 repetitive targets also enabled us to study the position dependence of mismatch tolerance by examining whether mismatches in certain positions are enriched in the off-target results versus the genomic background. In particular, the *DNMT1* repetitive guide has nearly 37,000 off-targets with a single mismatch to the on-target sequence and a TTTN PAM, according to Cas-OFFinder^24^. Each mismatched position is represented in at least 150 genomic loci, although the prevalence of a mismatch at a given target position is not uniformly distributed (Fig. 3b,c). Cpf1-BLISS detected ∼1,000 and ∼3,600 off-targets for AsCpf1 and LbCpf1, respectively, which contain only one mismatch to the on-target sequence. The fraction of Cpf1-BLISS-detected sites over all possible mismatches at that position was calculated to obtain a measure of how permissive Cpf1 is to mismatches along the guide (Fig. 3c). We also systematically introduced mismatches between the Cpf1 guide and target DNA, normalizing the on-target modification rate for each mismatched guide to the matched target (Fig. 3d and Supplementary Fig. 11). The on-target indel data from the mismatched guides were used to generate a composite model of the mismatch tolerance versus position for AsCpf1 and LbCpf1, with the overlaid SpCas9 trace based on reanalysis of previous mismatch data^27^ (Fig. 3e and Methods). Taken together, there appears to be three regions of the guide for both AsCpf1 and LbCpf1 where mismatches are more tolerated: (1) at the 3′-PAM distal end of the guide (positions 19–20); (2) towards the middle of the guide (positions 8–11); and, to a lesser degree, (3) at the first base at the 5′-PAM proximal end (position 1). This qualitatively suggests that Cpf1 may have several distinct regions of the guide that enforce complementarity and thereby contribute to its heightened specificity compared with SpCas9.

## Discussion

We developed a versatile, sensitive and quantitative method for direct genome-wide DSB profiling that is applicable to low-input samples of both cells and tissue, and is easily scalable for high-throughput DSB mapping in many samples. BLISS offers several unique features and advantages compared with the existing methods for genome-wide DSB detection: (1) robust discrimination of DSB events that occurred at the same genomic location in multiple cells or alleles, by using UMIs to filter out PCR duplicates; (2) applicability to low-input samples of cells and tissue sections, by performing all *in situ* reactions and washes on a solid surface; (3) assay scalability and cost-effective multiplexing by performing *in situ* reactions inside multi-well plates and barcoding samples in different wells before pooling; and (4) fast turnaround time compared with BLESS (∼12 active work-hours over 5 days to process 24 samples by BLISS versus at least 60 active work-hours over 15 days by BLESS). In addition, we demonstrate that BLISS is a highly sensitive method to assess the specificity of CRISPR-associated RNA-guided DNA endonucleases Cas9 and Cpf1, and we show that, in agreement with previous reports^25^,^26^, Cpf1 can provide high levels of editing specificity. In conclusion, BLISS is a powerful and versatile method for genome-wide DSB profiling that we believe will catalyse efforts to profile natural and artificially induced DSBs in many conditions and sample types.

## Methods

### Cells and tissues

The following cell lines were used: KBM7 from Oscar Fernandez-Capetillo (SciLifeLab, Stockholm, Sweden); U2OS from Mats Nilsson (SciLifeLab); HEK 293 from ATCC (although this cell line is catalogued as a commonly misidentified cell line in the ICLAC database (http://iclac.org/databases/cross-contaminations), we used it for CRISPR experiments, as it is easy to culture and can be efficiently transfected); mESCs from Simon Elsaesser (SciLifeLab). None of the cell lines was authenticated. Culturing conditions were as following: KBM7 in Iscove's modified Dulbecco's medium (Life Technologies, catalogue number 10829018), supplemented with 10% fetal bovine serum (FBS, Gibco, catalogue number F2442); U2OS in DMEM medium (Life Technologies, catalogue number D0819), supplemented with 10% FBS; HEK 293 T in DMEM supplemented with 10% FBS; and mESCs in minimal essential medium (Sigma, catalogue number M2279), supplemented with 20% FBS, 1% GlutaMAX (Gibco, catalogue number 35050061), 1% non-essential amino acids (Gibco, catalogue number 11140035), 1% sodium pyruvate (Gibco, catalogue number 11360070) and 0.2% β-mercaptoethanol, in the presence of leukaemia inhibitory factor (Sigma catalogue number L5158-5UG) corresponding to 1,000 U ml^–1^. All cell lines were tested to be mycoplasma free using MycoAlert Mycoplasma Detection Kit (Lonza, catalogue number LT07-118). For tissue-BLISS on mouse liver, wild-type, 6-week-old C57/BL6 male mice were killed following the guidelines in the MIT protocol 0414-027-17 ‘Modeling and Treating Genetic Disease Using Targeted Genome Engineering' (IACUC AWA A3125-01, IACUC 0411-040-14, approval date 5/16/2013).

### Cas or Cpf1 expression constructs and transfections

The selected targets for Cas9-BLISS are located within the *EMX1* locus (5′-GAGTCCGAGCAGAAGAAGAAgGG-3′) and the *VEGFA* gene locus (5′-GGTGAGTGAGTGTGTGCGTG tGG-3′). The plasmids used containing the SpCas9 and the sgRNA cassette were identical to the ones used for Cas9-BLESS^11^, where the targets were labelled as *EMX1*(1) and *VEGFA*(1). The same targets have also been studied using GUIDEseq^12^, where they were labelled as *EMX1* and *VEGFA*_site3. AsCpf1 and LbCpf1 along with their cognate CRISPR RNAs were cloned into the same expression vector as Cas9, to enable a direct comparison. Cells were plated before transfection in 24-well plates pre-coated with poly-D-lysine (Merck Millipore, catalogue number A003E) at a density of ∼125,000 per well and were let grow for 16–18 h until 60–70% confluence. For transfections, we used 2 μl of Lipofectamine 2000 (Life Technologies, catalogue number 11668019) and 500 ng of Cas9 plasmid in 100 μl total of OptiMEM (Gibco, catalogue number 31985062) per each well of a 24-well plate.

### Immunofluorescence staining

γH2A.X immunostaining was performed using a mouse anti-phospho-histone H2A.X (ser139) primary antibody (Millipore, catalogue number 05-636) diluted 1:1,000 in blocking buffer and a goat anti-mouse IgG (H+L) Alexa Fluor 647 conjugate (Thermo, catalogue number A-21235) secondary antibody diluted 1:1,000 in blocking buffer. To image γH2A.X foci, we acquired images every 0.4 μm throughout the entire nuclear volume using a × 40 oil objective and an LSM 780 confocal microscope (Zeiss).

### BLISS adapters

All BLISS adapters were prepared by annealing two complementary oligonucleotides as described below. All oligos were purchased from Integrated DNA Technologies as standard desalted oligos. UMIs were generated by random incorporation of the four standard dNTPs using the ‘Machine mixing' option. Before annealing, sense oligos diluted at 10 μM in nuclease-free water were phosphorylated for 1 h at 37 °C with 0.2 U μl^–1^ of T4 Polynucleotide Kinase (NEB, catalogue number M0201). Phosphorylated sense oligos were annealed with the corresponding antisense oligos pre-diluted at 10 μM in nuclease-free water, by incubating them for 5 min at 95 °C, followed by gradual cooling down to 25 °C over a period of 45 min (1.55 °C min^−1^) in a PCR thermocycler.

### BLISS sample preparation

A step-by-step BLISS protocol is provided in Protocol Exchange^18^. For BLISS in cell lines, we typically either grew cells directly onto 13 mm coverslips (VWR, catalogue number 631-0148) or we spotted them onto coverslips pre-coated with poly-L-lysine (Sigma, catalogue number P8920-100ML). For Cas9 and Cpf1 experiments, we fixed HEK293T cells directly into the 24-well plate used for transfections and performed all *in situ* reactions directly inside the wells of the plate. For BLISS in mouse liver, we developed two approaches: (1) Tissue cryopreservation and sectioning: freshly extracted liver biopsies were first fixed in paraformaldehyde 4% for 1 h at 25 °C and then immersed in a sucrose solution (15% overnight and then 30% until the tissue sank) before embedding in optimal cutting temperature medium (OCT). Thirty-micrometre-thick tissue sections were mounted onto microscope slides, dried for 60 min at room temperature (rt) and stored at 4 °C before further processing. (2) Preparation of nuclei suspensions: freshly extracted liver biopsies were cut into small pieces and transferred into a 1.5–2 ml tube containing nucleus isolation buffer (NaCl 146 mM, Tris-HCl 10 mM, CaCl_2_ 1 mM, MgCl_2_ 21 mM, bovine serum albumin 0.05%, Nonidet P-40 0.2% pH 7.8). We typically incubated the samples for 15–40 min until the tissue fragments became transparent, after which the nuclei were centrifuged for 5 min at 500 *g* and then re-suspended in 200–500 μl of 1 × PBS. One hundred microlitres of nuclei suspension were dispensed onto a 13 mm diameter poly-L-lysine-coated coverslip and incubated for 10 min at rt. Afterwards, 100 μl of paraformaldehyde 8% in 1 × PBS were gently added and incubated for 10 min at rt, followed by two washes in 1 × PBS at rt. The samples were stored in 1 × PBS at 4 °C up to 1 month before performing BLISS.

### *In situ* DNA digestion

Samples for DNA accessibility mapping were prepared in the same way as BLISS samples, except that the *in situ* DSBs blunting step was substituted by an *in situ* DNA digestion step using 1 U μl^–1^ of HindIII endonuclease (NEB, catalogue number R3104) and incubating the samples for 18 h at 37 °C. HindIII cut sites were ligated with modified BLISS adapters carrying the HindIII complementary sticky end (see Supplementary Fig. 4h). To prevent *in situ* re-ligation of HindIII cut sites, the samples were incubated for 2 h at 37 °C in the presence of 0.015 U μl^–1^ of calf intestinal alkaline phosphatase (Promega, catalogue number M2825) before *in situ* ligation.

### Image processing and counting of γH2AX foci and cells

All algorithms were implemented in MATLAB using custom-made scripts, available upon request. To count γH2AX foci in KBM7 cells, we first segmented nuclei stained with 4,6-diamidino-2-phenylindole using image thresholding. We then identified all local maxima within each image and then ranked the maxima according to their response to a Laplacian filter. We then fitted a Gaussian to the first peak of the histogram of the filter responses, corresponding to background noise (that is, autofluorescence and photon noise). We counted γH2AX foci per nucleus using the dots with a filter response of more than 10 s.d. above the mean of the background. To count cells before capture and gDNA extraction, we first rinsed samples in nuclease-free water, air dried them and acquired wide-field images of areas selected for cell capture using a TI-S-E Motorized stage operated by NIS-Elements software (Nikon). Next, we identified objects in wide-field images by locating maxima of the determinant of the gradient structure tensor. We then classified objects being cells or not based on anisotropy, size and median gradient magnitude. Finally, we manually corrected and verified the segmentation.

### Pre-processing of sequencing data

To convert the raw sequencing data into BED files ready to be used for *ad ho*c analyses, we applied the pipeline summarized in Supplementary Fig. 1e. Briefly, we filtered the FASTQ files for overall quality by requiring a Phred score ≥30 for every base. Thereafter, we scanned the filtered reads for the presence of the exact prefix (8N UMI and sample barcode), by allowing up to two mismatches in the UMI portion and up to one mismatch in the barcode (see analysis of UMI errors below). After removal of the prefix, we aligned the reads to the reference genome (GRCh37/hg19 for human, NCBI37/mm9 for mouse). We retained reads mapping with a quality score ≥5, after excluding regions with poor mappability. Next, we performed a further filtering step based on UMI sequences to filter out PCR duplicates. Reads mapping in nearby locations (at most 8 nt apart) and having at most two mismatches in the UMI sequence were associated with the location of the most frequent read in the neighbourhood. Finally, we generated BED files containing a list of genomic locations associated with unique UMIs to be used in downstream analyses.

### UMI error model

In BLISS, the incorporation of UMIs at the site of *in situ* DSB ligation enables distinguishing breaks occurring at the same nucleotide position in different alleles or cells. However, during amplification by *in vitro* transcription and PCR, as well as during sequencing, the original UMI sequence may be subject to errors that in turn can cause both false positive (the same DSB labelled by two different UMIs) and false negative (two distinct DSB events labelled by the same UMI) errors. It is therefore important to implement an error-correction scheme that aims to maximize the number of unique DSB events identified, while minimizing the number of false-positive DSB callings. To do so, we first performed an experiment in which we *in situ* digested gDNA using a restriction enzyme (HindIII), followed by *in situ* ligation of the modified BLISS adapter shown in Supplementary Fig. 1f. Thus, in this experiment, R1 reads are expected to start with the 8 nt fixed UMI sequence, 5′-GTCGTCGC-3′ followed by the 6 nt HindIII recognition sequence, 5′-AAGCTT-3′. To assess the error rates associated with amplification and sequencing, we considered for simplicity only mismatch errors. We first filtered the FASTQ file by selecting all the strings of 8 bp found before the AAGCTT sequence (allowing for 1 mismatch). Then, we counted how many of these strings contain up to eight mismatches in the fixed UMI sequence. As shown in Supplementary Fig. 1g, most reads (∼77%) had 0 mismatches in the UMI sequence, whereas ∼16% had 1 mismatch. Importantly, grouping together faulty UMIs with 1 or 2 mismatches takes into account 90% of the mismatch errors, indicating that counting as distinct DSB ends the R1 reads that map to the same genomic location and tagged with UMIs differing for at least 2 nt is a reliable procedure.

To further corroborate these observations, we performed one additional experiment in which we *in vitro* transcribed a synthetic DNA fragment purchased from Integrated DNA Technologies as gBlocks Gene Fragments, containing (from 5′): the T7 promoter sequence; the Illumina RA5 adapter sequence; the 16 nt sequence 5′-GTCGTATCGTCGTTCC-3′ representing a ‘fixed' UMI, the HindIII cutting site 5′-AAGCTT-3′ and 469 nt taken from the ampicillin resistance open reading frame. Out of 1,900,898 reads obtained, 1,274,568 (67%) had at most 1 mismatch in the HindIII recognition site location and were preceded by 16 nt, as expected. Of these, 1,273,545 (99.9%) reads had at most 1 mismatch in the 8 nt preceding the cut site. Therefore, by filtering the initial FASTQ file for a prefix of the form UMI-barcode[1,0,0]-cutsite[1,0,0] (numbers in square brackets indicate the allowed number of mismatches, insertions and deletions, respectively), we might lose at most 30% of the sequenced reads. This percentage is not significantly lowered by allowing for more mismatches in the cutsite location or in the barcode location. We note that taking into account small insertions and deletions (indels) might reduce the number of reads filtered out. However, accounting for indels in the error model would make downstream read identification more ambiguous. Hence, we decided to stick to an error model that is more stringent, but more robust to false positive errors. In conclusion, for all the data sets presented in the paper, we filtered FASTQ files based on the prefix: UMI[2,0,0]-barcode.

### Identification of telomeric ends

To analyse the composition of BLISS reads derived from the telomeric C-rich strand, we screened R1 reads with the correct prefix (8N UMI and sample barcode) for the presence of each of the six possible patterns based on the human telomeric sequence: [#A,#AA,#TAA,#CTAA,#CCTAA,#CCCTAA]-CCCTAA.

### Estimation of DSBs per cell

To estimate the number of spontaneous DSBs, we sequenced at different depth three libraries prepared from small numbers of KBM7 cells (L1, L3 and L4, see Supplementary Data 2). For each sample, we estimated the number of DSBs per cell by counting the number of sequenced reads with correct prefix mapped to a unique genomic location and tagged by a unique UMI and assuming that on average one DSB produces two unique reads. We then fitted the data to the model 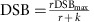, where DSB_max_ is the number of DSB events per cell at saturation, *r* is the number of total reads and *k* is a constant. At saturation, the model estimated DSB_max_=94 breaks per cell (95% confidence interval: 93.10–95.07), in agreement with γH2A.X foci counting in the same cell line (Supplementary Fig. 2c).

### Quantification of etoposide effects

For U2OS cells treated with etoposide, we counted the number *n* of unique DSB locations on each chromosome that were found with at least 1≤*t*≤10 UMIs and at most *t*=500 UMIs. We then normalized the cumulative sum, *n* by the total number of DSB ends sequenced and calculated the ratio between the normalized cumulative sum in the treated and non-treated sample, and averaged the fold change over all chromosomes. We repeated the same process separately for the unique locations exclusively found in the etoposide-treated or untreated sample. For enrichment analysis of etoposide-induced DSBs around the TSS, we calculated the fraction of unique DSB locations (found with at least 1≤*t*≤10 UMIs and at most *t*=500 UMIs) that fell in a window of ±5 kb centred on the TSS of all genes.

### Quantification of DSBs near TSS and within gene bodies

For mouse liver and mESCs, we used RNA-seq data obtained from the Mouse Encode Project at Ren lab (http://chromosome.sdsc.edu/mouse/download.html). We first identified the top 10% and bottom 10% expressed genes and then, for each gene in the two groups, we calculated the number of unique DSB ends (that is, the number of DSB locations on either strand associated with a unique UMI) falling in an interval of ±5 kb centred on the TSS of the gene. This approach enabled us to distinguish DSBs that had occurred at the same genomic location in different cells. We then calculated the proportion of all the DSB locations mapped around the TSS of both top 10% and bottom 10% expressed genes, that fell in a given distance interval near the TSS. For gene bodies, we performed a similar analysis by counting all the unique DSB ends mapped within the gene body of the top 10% and bottom 10% expressed genes, and normalizing the counts by gene length.

### Gene ontology analysis of top fragile genes

We identified top 10% fragile genes in three biological replicates of mouse liver tissue sections either based on the number of unique DSB ends mapped in a ±1 kb interval centered on the TSS of all genes or based on the number of unique DSB ends mapped within the gene bodies. We performed GO process analysis of the fragile genes identified in all the three biological replicates, using the publicly available web-based Gorilla tool (http://bmcbioinformatics.biomedcentral.com/articles/10.1186/1471-2105-10-48).

### Identification of Cas9 and Cpf1 on- and off-target DSBs

We updated the original DSB detection pipeline for analysing Cas9-BLESS data^9^,^10^ to determine whether we could enhance the sensitivity of off-target detection by both BLESS and BLISS. Previously, we demonstrated that a homology search algorithm was capable of separating *bona fide* Cas9-induced DSBs from background DSBs and performed the analysis on the top 200 DSB loci with the strongest signal after initial filtering^10^,^11^. To achieve even greater sensitivity, here we extended this homology search to the top 5,000 DSB locations identified by BLISS. To enable a direct comparison between BLESS and BLISS, we used this updated approach to re-analyse the BLESS data previously obtained with wild-type SpCas9 (ref. 11) on the same *EMX* and *VEGFA* guide targets as studied here. Briefly, a ‘Guide Homology Score' was determined using an algorithm that searched for the best-matched guide sequence within a region of the genome 50 nt on either side of the centre of a DSB cluster identified in BLESS/BLISS for all NGG and NAG PAM sequences in the case of SpCas9 (ref. 11) and all possible PAMs in the case of AsCpf1 and LbCpf1 for maximum sensitivity. A score based on the homology was calculated using the Pairwise2 module in the Biopython Python package with the following weights: a match between the sgRNA and the genomic sequence scores +3, a mismatch is −1, whereas an insertion or deletion between the sgRNA and genomic sequence costs −5. Thereby, an on-target sequence with the fully matched 20 bp guide would have a Guide Homology Score of 60. Previously, we included the PAM match in the scoring, yielding a maximum score of 69, but to make the score more versatile and comparable across different PAMs, we removed the PAM dependence in the scoring. Using this guide homology score, we performed a receiver operating characteristic curve analysis based on validated and non-validated off-targets from SpCas9-BLESS^10^, which justified our previous choice of a homology score cutoff (41 out of a max score of 60), to maximize the sensitivity and specificity of Cas9-BLISS and Cpf1-BLISS. In practical terms, this score corresponds to ≤4 mismatches or ≤2 gaps, as well as combinations thereof.

### Modelling mismatch tolerance per-position of Cas9/Cpf1

Analysis of tolerance to mismatches at different positions along target/sgRNA duplex. The cutting frequency of Cas9/Cpf1 at a target with a single mismatch is modelled as


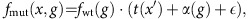


where 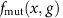 represents the cutting frequency with sgRNA *g* at the target that has a mismatch to the sgRNA at position *x*, 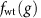 represents the cutting frequency with sgRNA *g* at its perfectly matching target, *t*(*x*′) denotes the tolerance of mismatch at position *x*′, *a*(*g*) represents the effect of sgRNA *g* specific properties on the mismatch tolerance (properties such as transfection efficiency, melting temperature, secondary structure and so on) and 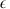 represents experimental variation. The position *x* on the sgRNA may, in reality, shift to position *x*′ due to stretch or compression of the sgRNA-target DNA hetero-duplex. The amount of shift can be different for different sgRNA, mismatch pairing and positions. This effect is termed ‘wobbling'. Given the measured cutting frequency 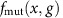 and 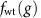, we are interested in recovering *t*(*x*), which models the position-dependent mismatch tolerance, a property of the Cas9 and Cpf1 protein that is independent of sgRNA sequences, target or transfection batches. We solve the following problem,


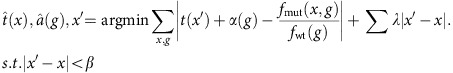


The *t*(*x*) is modelled using a third-order B-spline, a continuously differentiable function defined on interval (1,20) and 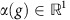. The optimization is solved using gradient descent. The optimal solution 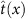 is normalized to get 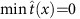 and 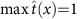. The parameter *λ* controls the strength of lasso, which is set to 0.3. The parameter *β* represents the range of wobbling, which is set to 0.5.

### Data availability

All sequencing data related to this study have been deposited in the NCBI Sequence Read Archive at SRP099132. All other data are available from the authors upon reasonable request.

## Additional information

**How to cite this article:** Yan, W. X. *et al*. BLISS is a versatile and quantitative method for genome-wide profiling of DNA double-strand breaks. *Nat. Commun.* **8,** 15058 doi: 10.1038/ncomms15058 (2017).

**Publisher's note**: Springer Nature remains neutral with regard to jurisdictional claims in published maps and institutional affiliations.

## Supplementary Material

Supplementary Information

Supplementary Data 1 Excel table containing the list of oligo adapters used in the paper. Upper and bottom oligos have the structure of the top and bottom oligos depicted in Supplementary Fig. 1a.

Supplementary Data 2 Excel table containing a summary of sequencing data. SE, single-17 end sequencing. PE, paired-end sequencing

Supplementary Data 3 Excel table containing the list of top 10% fragile genes identified by BLISS in three biological replicates of mouse liver tissue sections. Fragile genes were defined either based on the 90th percentile of the number of sequenced DSB ends mapped ±1 kb around TSS or based on the 90th percentile of the number of sequenced DSB ends per kilobase mapped inside gene bodies. Shared genes represent fragile genes identified in all three replicates.

Supplementary Data 4 Excel table containing the list of GO function terms significantly enriched in the top 10% fragile genes identified in all the three biological replicates of mouse liver tissue sections. P value, enrichment P value computed according to the hypergeometric model. FDR q-value, correction of the P value for multiple testing using the BenjaminiHochberg method. N, total number of mouse genes. B, total number of genes associated with a specific GO term. n, top 10% fragile genes. b, number of genes in the intersection. Enrichment is calculated as (b/n) / (B/N).

Supplementary Data 5 Excel table containing the list of on- and off-target Cas9 and Cpf1 sites identified by BLISS. Sheet #1 contains the list of Cas9 targets of EMX1 and VEGFA sgRNAs examined in benchmarking BLISS with GUIDEseq and Digenome-seq. Sheet #2 contains the list of targets of sgRNAs examined in assessing AsCpf1 and LbCpf1 specificity and comparing these nucleases to SpCas9 and eSpCas9.
Double-strand breaks are a major DNA lesion 1 that can occur by endogenous and exogenous processes. Here the authors present BLISS — Breaks Labelling In Situ and Sequencing — to map breaks across the genome.
