# Supplementary material for: BLISS is a versatile and quantitative method for genome-wide profiling of DNA double-strand breaks
Source: Nat Commun. 2017 May 12;8:15058. doi: 10.1038/ncomms15058 (PMC5437291; doi:10.1038/ncomms15058)
Supplement: Supplementary Information [file ncomms15058-s1.pdf]

SUPPLEMENTARY FIGURES

Supplementary Figure 1

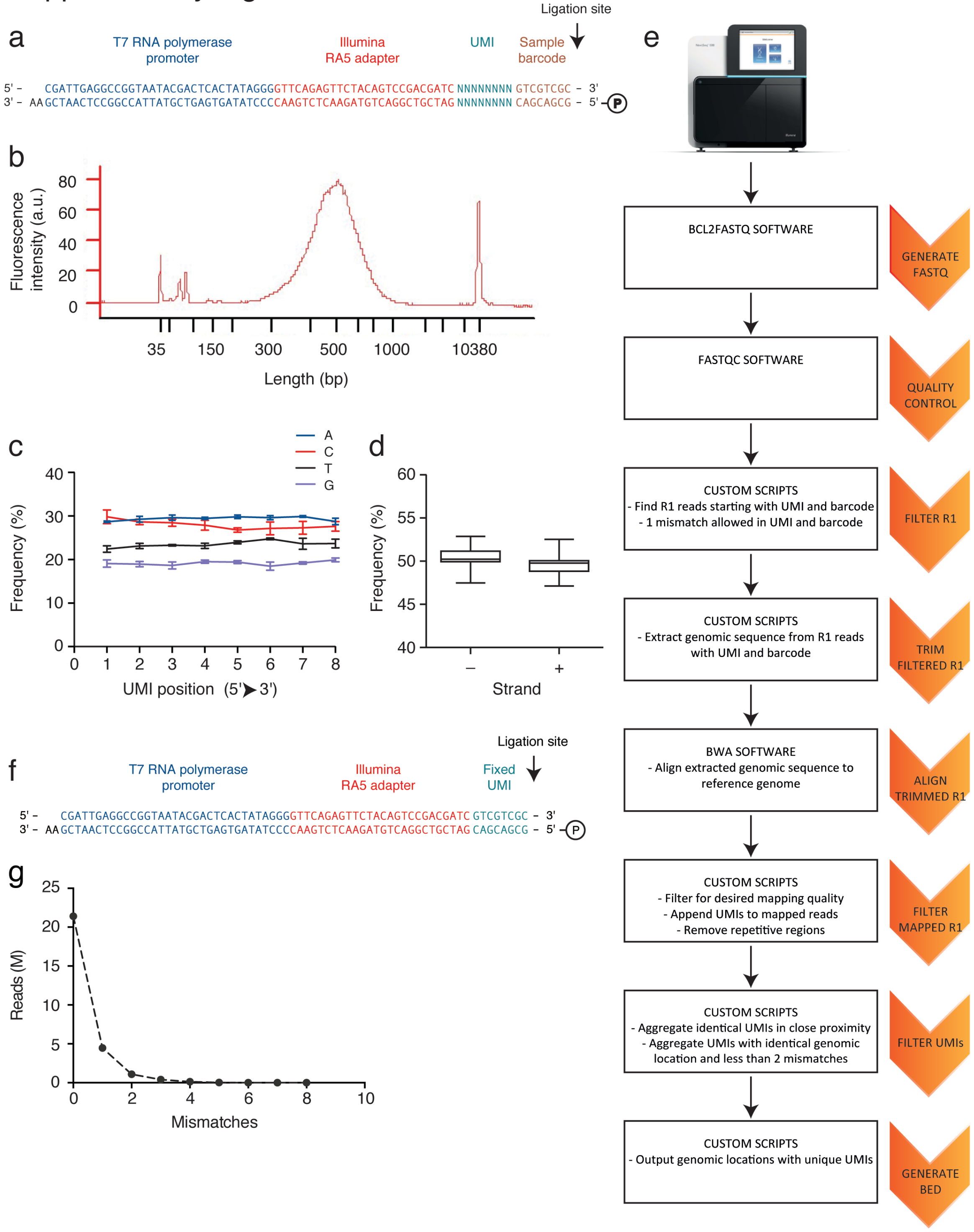

Supplementary Figure 1. (**a**) Structure of BLISS adapters. The bottom oligo terminates with a 3′ overhang to prevent the formation of head-to-tail concatemers. Ligation to free blunted DSB ends occurs at the site marked by the black arrow. The 5′-phosphate group is either

added directly during oligo synthesis or using T4 polynucleotide kinase. Annealing of the upper and bottom oligo is performed as described in **Experimental Methods**. A full list of BLISS adapter sequences used in this paper is provided in **Supplementary Data 1**. (**b**) Bioanalyzer plot showing the typical fragment size distribution of a good BLISS library. (**c**) Percentage of each DNA base at every nucleotide position in sequenced UMIs. Dots, mean value. Whiskers, ±s.d. The deviation of frequencies from theoretical 25% is due to biases in the rate of base incorporation during oligo synthesis (Integrated DNA Technologies Inc., personal communication). (**d**) Percentage of reads mapped to the minus versus plus strand. Whiskers, min–max range. (**e**) General workflow for pre-processing BLISS sequencing data. All steps were implemented using custom pipelines in Unix that can be provided upon request. (**f**) Modified BLISS adapter used to calculate the frequency of errors inside the UMI region. (**g**) Frequency of reads carrying one or more mismatches inside the UMI region.

Supplementary Figure 2

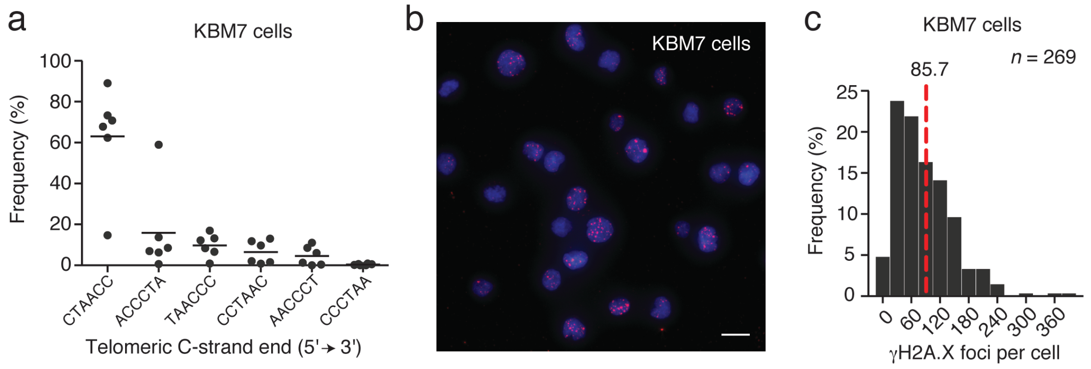

Supplementary Figure 2. (**a**) Percentage of each of the six possible starting frames of 5′ telomeric ends. (**b**) γH2A.X foci (red) in the nucleus (blue) of KBM7 cells. Z-projection of maximum intensity is shown. Scale bar, 20 μm. (**c**) Distribution of the number of γH2A.X foci per cells. *n*, number of cells analyzed. The number near the dashed red line equals the mean number of γH2A.X foci per cell.

Supplementary Figure 3

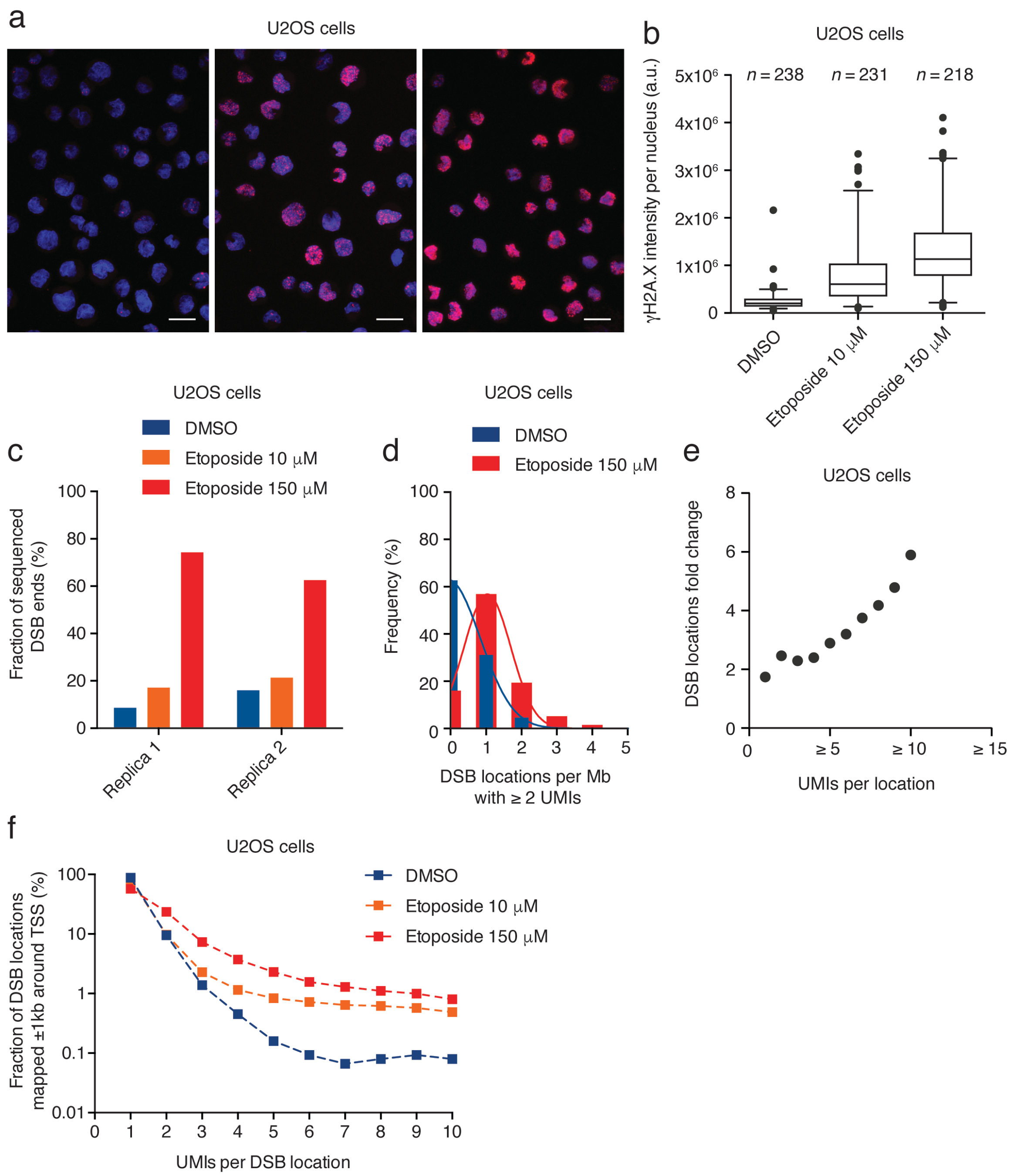

Supplementary Figure 3. (**a**) γH2A.X signal (red) in the nucleus (blue) of control versus etoposide-treated U2OS cells. Scale bars, 20 μm. (**b**) Nuclear intensity of γH2A.X in control versus etoposide-treated U2OS cells. *n*, number of cells analyzed. Whiskers extend from 2.5 to 97.5 percentiles. (**c**) Percentage of sequenced DSB ends in control versus etoposide-treated U2OS cells. Two biological replicas were analyzed. For each condition, the same amount of genomic DNA was loaded into a single IVT reaction, and a single sequencing library was

prepared for each replica. (**d**) Percentage of DSB locations associated with at least two UMIs inside a 1 Mb genomic interval, in control versus etoposide-treated U2OS cells. Continuous lines, Gaussian interpolation. (**e**) Difference (fold change) in the number of DSB locations associated with at least the indicated number of UMIs, in etoposide-treated versus control U2OS cells. (**f**) Number of DSB locations in etoposide-treated versus control U2OS cells by filtering on the minimum number of UMIs per DSB location.

Supplementary Figure 4

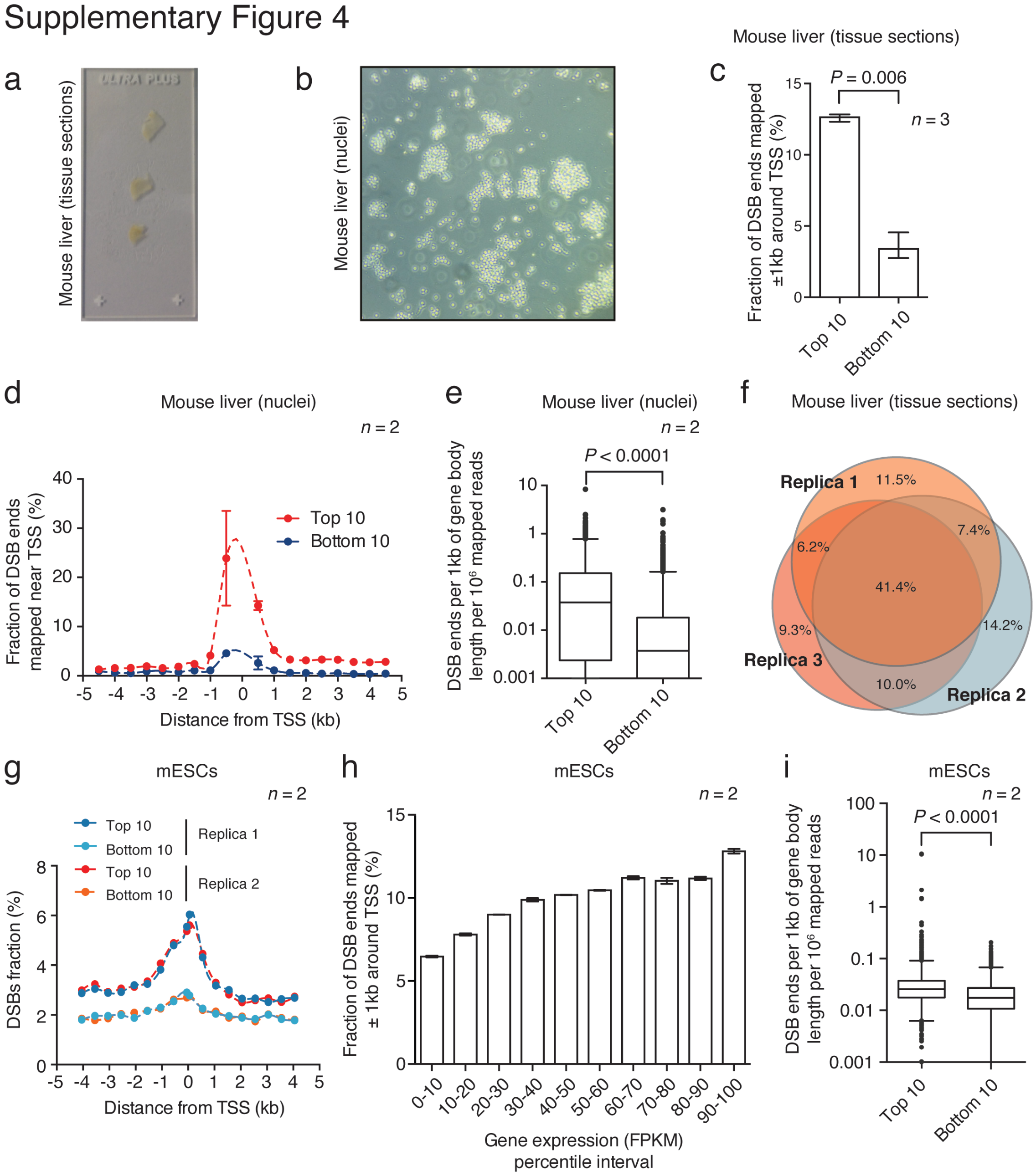

Supplementary Figure 4. (**a**) Bright-field image of mouse liver fixed tissue sections mounted onto a microscope slide and ready to be processed by BLISS. (**b**) Bright-field image of mouse liver fixed nuclei attached onto a poly-D-lysine coated coverslip and ready to be processed by BLISS. (**c**) Percentage of sequenced DSB ends mapped ±1 kb around the TSS of top 10% versus bottom 10% expressed genes in mouse liver. *n*, number of biological replicates. Bars, mean value. Whiskers, range. *P*, paired t test. (**d**) Percentage of sequenced DSB ends mapped ±4.5 kb around the TSS of the top 10% and bottom 10% expressed genes in mouse liver. *n*, number of biological replicates. Dots, mean value. Whiskers, range. Dashed lines, spline interpolation. (**e**) Number of sequenced DSB ends mapped per kilobase inside the gene body of the top 10% and bottom 10% expressed genes in mouse liver. *n*, number of

biological replicates. Whiskers, 2.5–97.5 percentile range. *P*, Mann-Whitney test. (**f**) Overlap between top 10% fragile genes identified in three mouse liver biological replicates based on the number of sequenced DSB ends mapped ±1 kb around the TSS. (**g**) Percentage of sequenced DSB ends mapped ±4.5 kb around the TSS of the top 10% and bottom 10% expressed genes in two biological replicates of mouse embryonic stem cells. Dashed lines, spline interpolation. (**h**) Percentage of sequenced DSB ends mapped ±1 kb around the TSS for each inter-decile interval of gene expression in mouse embryonic stem cells. FPKM, fragments per kilobase of transcript per million mapped reads. *n*, number of biological replicates. Bars, mean value. Whiskers, min–max range. (**i**) Number of sequenced DSB ends mapped per kilobase inside the gene body of the top 10% and bottom 10% expressed genes in mouse embryonic stem cells. *n*, number of biological replicates. Whiskers, 2.5–97.5 percentile range. *P*, Mann-Whitney test.

Supplementary Figure 5

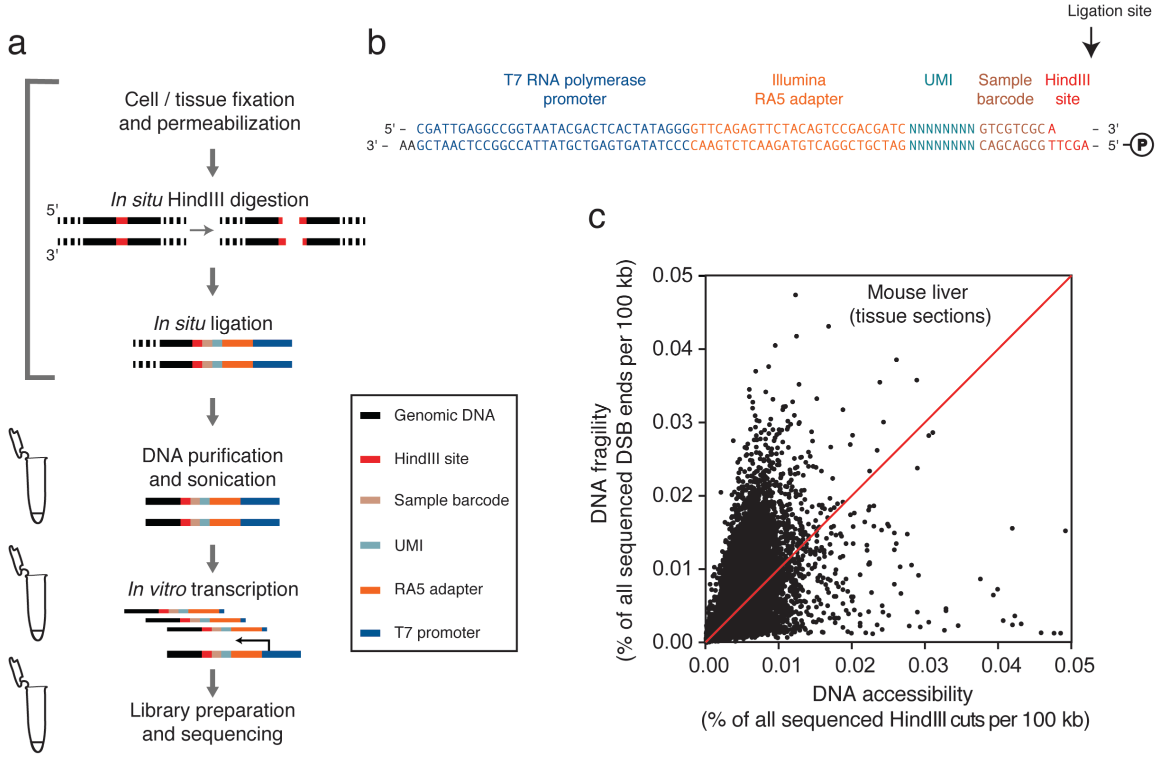

Supplementary Figure 5. (**a**) Modified BLISS workflow for measuring DNA accessibility. A restriction enzyme is applied *in situ* to induce artificial DSBs with sticky ends. The breaks are then *in situ* ligated using a modified BLISS adapter. The remaining steps are the same as in BLISS. In principle, any type of restriction endonuclease that produces sticky ends and has a known cutting pattern along the genome can be used. (**b**) Structure of the modified BLISS adapter used for profiling chromatin accessibility *in situ*. (**c**) Density of HindIII-induced breaks and natural DSBs in 100 kb intervals along the mouse genome. Each dot represents a 100 kb interval. For each bin, the percentage of all breaks mapped inside it is shown.

Supplementary Figure 6

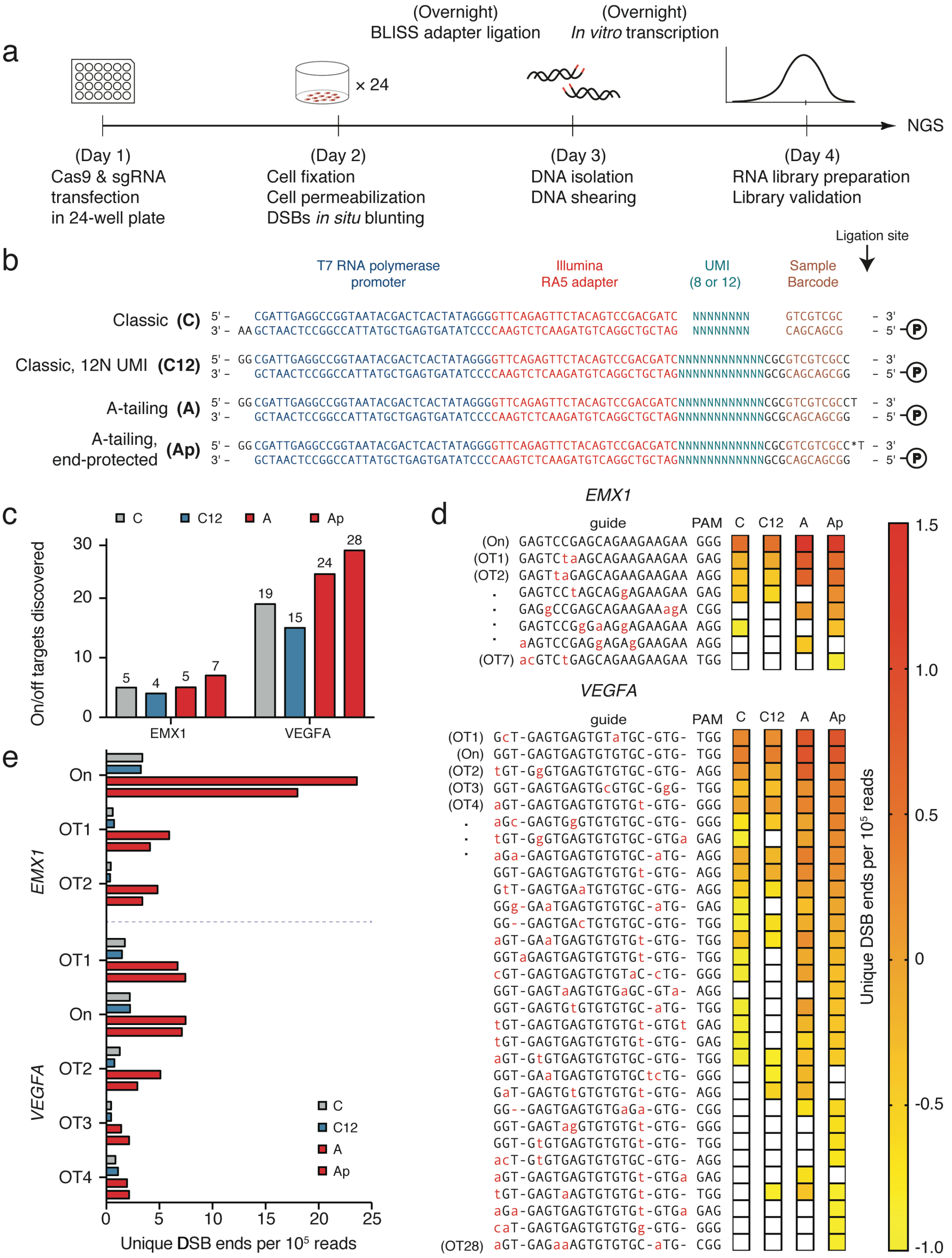

Supplementary Figure 6. (**a**) Scheme of Cas9-BLISS in 24-well plates. *In situ* DSB blunting and ligation reactions are performed directly in the multi-well plate. HEK 293T cells were used for transfection of sgRNA and SpCas9 constructs. (**b**) Scheme of different BLISS adapters used for optimizing Cas9 on- and off-target detection efficiency. The classic adapter is the same as in **Supplementary Fig. 1a**. A longer UMI was tested in order to increase the

number of unique on- and off-target break events that can be detected after PCR duplicate removal. P, 5′-phosphate group. (**c**) On- and off-target Cas9 DSB ends per 10^5 sequenced reads for each of the four adapters shown in (**b**) and two sgRNAs targeting the EMX1 and VEGFA genes. (**d**) Total number of DSB ends aligned to on- and off-targets in EMX1 and VEGFA genes per 10^5 sequenced reads for each of the BLISS adapters described in (**b**). (**c**) On- (ON) and off-targets (OFF) detected by BLISS using the four different adapters and ranked in descending order. (**e**) DSB score for the on- and top off-targets detected with different BLISS adapters. The end-protected, T-tailed adapter is more efficient in detecting the on- and top 2–3 off-targets.

Supplementary Figure 7

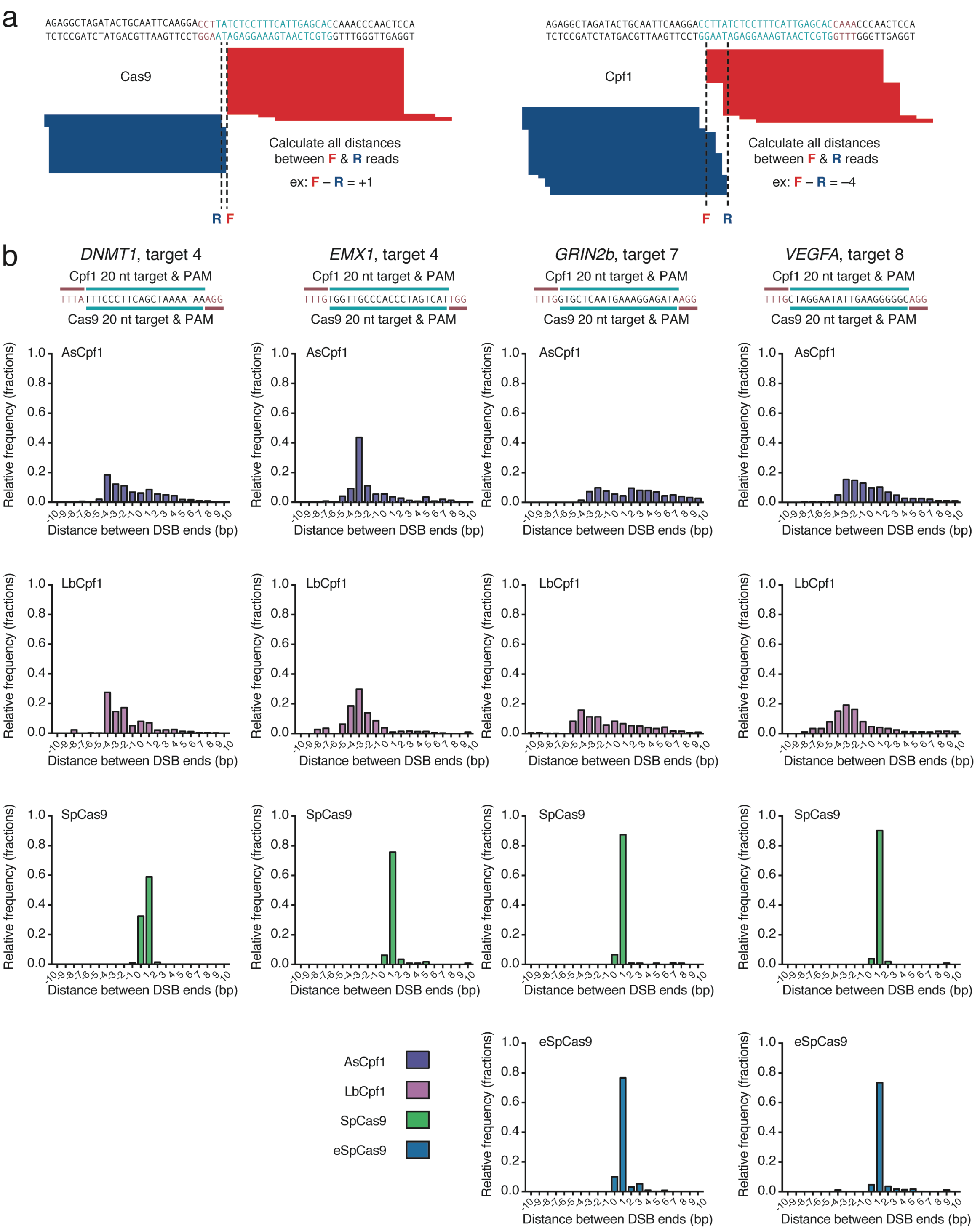

Supplementary Figure 7. (**a**) Representative DSB mapping and distance calculation at on-target sites for Cas9 and Cpf1 nuclease activity. Red indicates reads mapped to the positive strand, blue reads mapped to the negative strand. Distances were calculated between all positive and negative reads. The PAMs for the respective enzymes are labeled in magenta while the genomic target is in cyan. (**b**) Histograms of differences between positive and negative reads mapped for targets with concurrent AsCpf1, LbCpf1, SpCas9, and eSpCas9 targeting.

Supplementary Figure 8

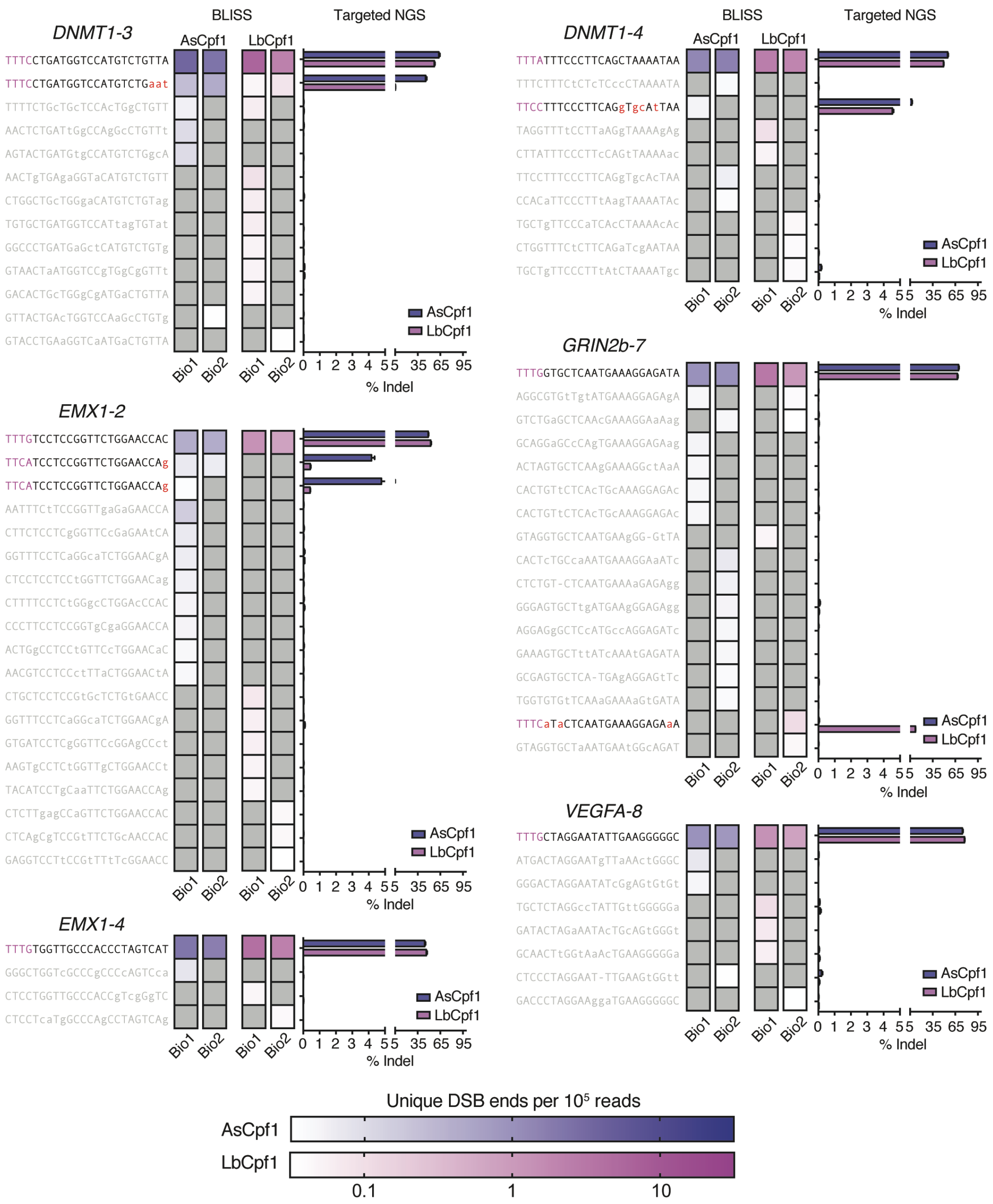

Supplementary Figure 8. Genome-wide characterization of AsCpf1 and LbCpf1 specificity using BLISS, with the presence of indels at the indicated targets are evaluated using targeted NGS. Off-targets were evaluated at loci from combined independent biological replicates and both AsCpf1 and LbCpf1 in order to maximize sensitivity. For the target sequence, the PAM is in magenta and mismatches to the on-target sequence are indicated in red. Grayed out sequences indicate loci in which no indels were observed above the negative control. (n = 3, error bars show s.e.m.).

Supplementary Figure 9

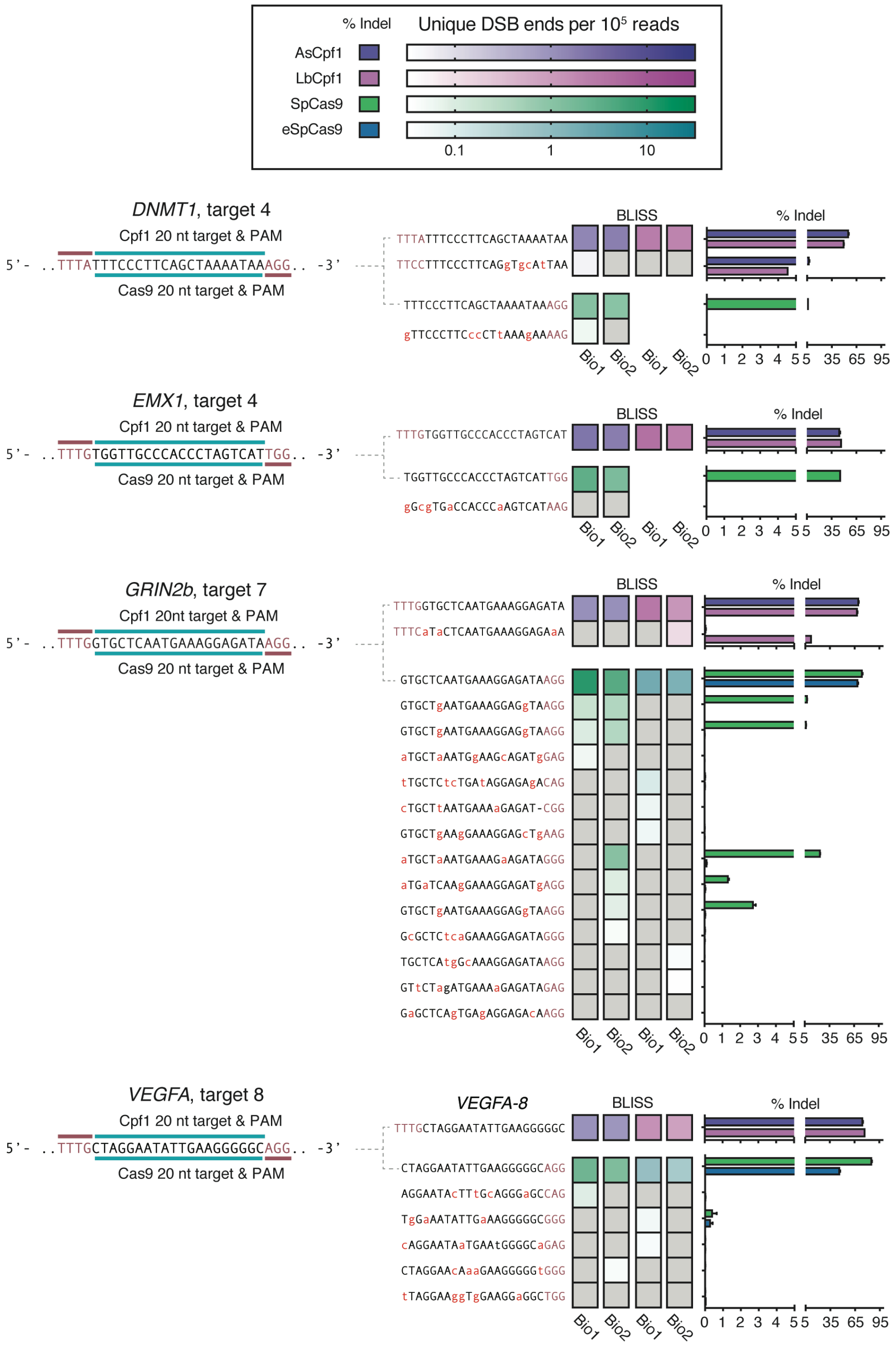

Supplementary Figure 9. Comparison of BLISS results at guides targeted by both AsCpf1, LbCpf1, SpCas9, and eSpCas9. Cpf1-BLISS data are redisplayed from **Figure 3a** for clarity of comparison.

Supplementary Figure 10

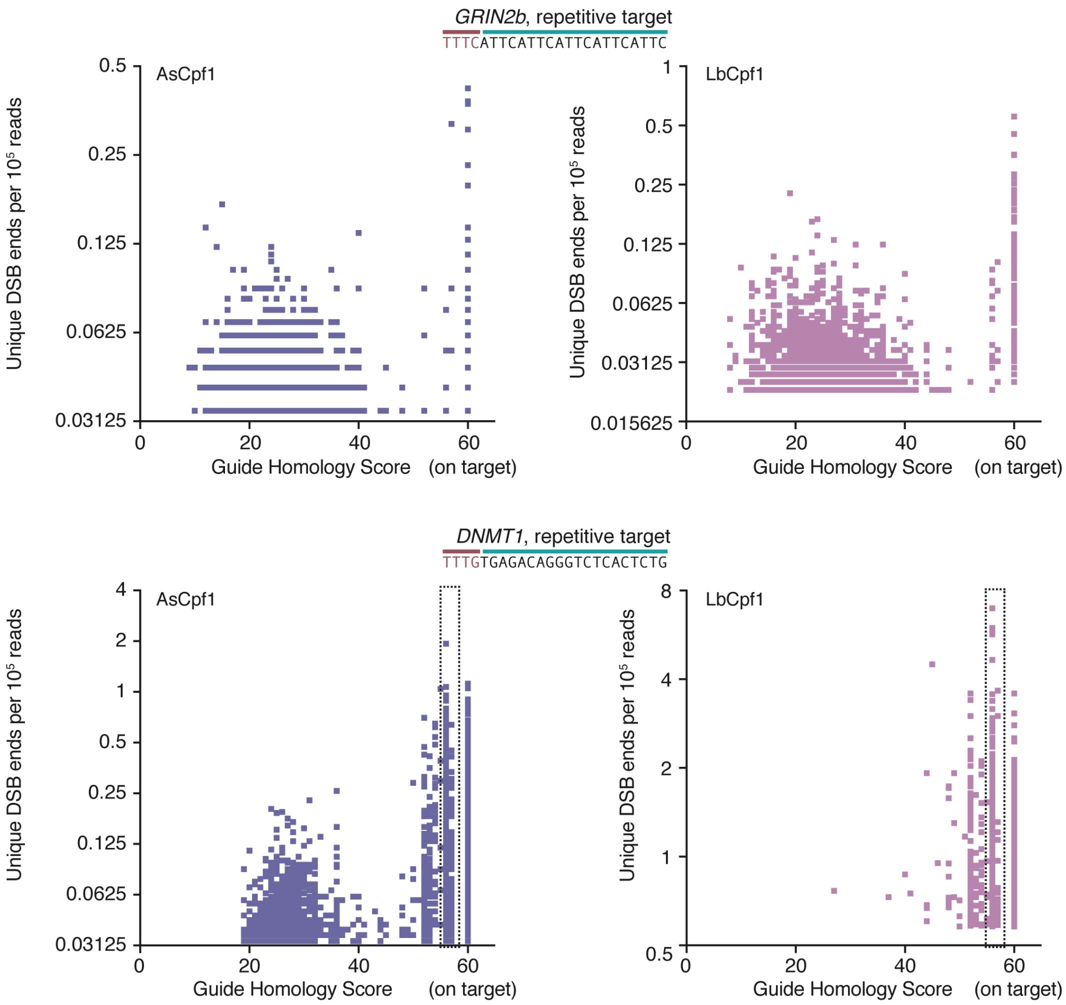

Supplementary Figure 10. Cpf1-BLISS for guides targeted to two repetitive sequences in the genome. Plot displays the guide homology score (for which 60 is a perfect match to the on-target sequence) versus the DSB score (a measure of unique DSBs at a particular locus). The outlined box indicates the BLISS-identified 1 bp mismatch genomic loci that were used in the position-dependent mismatch tolerance analysis in **Fig. 3c**.

Supplementary Figure 11

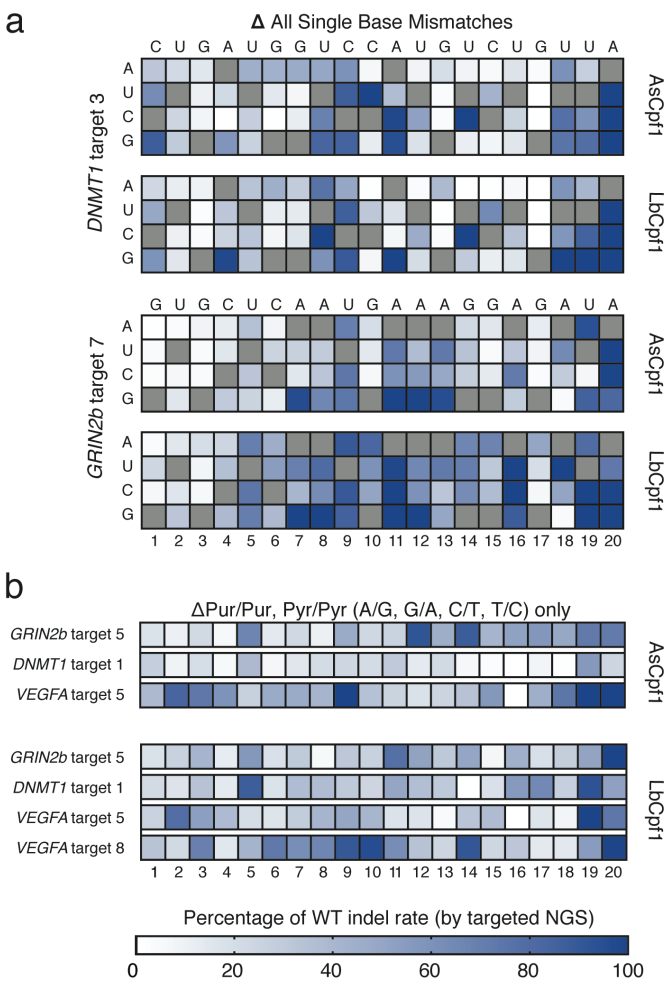

Supplementary Figure 11. Systematically investigating the tolerance of AsCpf1 and LbCpf1 to single base pair mismatches by measuring the fractional efficiency of on-target modification with guide RNAs which contain (**a**) all possible single base pair mismatches at each position along the 20 nt target sequence and (**b**) purine-to-purine and pyrimidine-to-pyrimidine mismatches. On-target indel rates are measured by targeted NGS and normalized to the fully matched target sequence. Values are the average of three technical replicates.

SUPPLEMENTARY TABLES

Supplementary Table 1. Comparison of BLISS with other methods for genome-wide DSB detection.

Method	Detection	Main features	Sample (input)	Reported applications
BLISS	Direct	*In situ* DSB blunting and ligation in cells and tissue sections attached onto a solid surface. Selective amplification of DSB ends by *in vitro* transcription. Quantitative power thanks to UMIs	Fixed cells, tissue sections (compatibility with low-input samples of 10^3 cells)	Etoposide-induced DSBs. Natural DSBs in cells and tissues. Cas9 and Cpf1 specificity (this paper)
BLESS	Direct	*In situ* DSB blunting and ligation of biotinylated adapters. DSB capture on streptavidin	Fixed cells (at least 10^6 cells)	Replication stress-induced DSBs in mammalian cells[1], Cas9 specificity[2,3]
DSBCapture	Direct	*In situ* DSB blunting and A-tailing. Modified BLESS adapters containing Illumina adapter sequences	Fixed cells (at least 10^6 cells)	DSBs at G-quadruplex rich sites, active genes and transcription start sites[4]
End-Seq	Direct	*In vivo* DSB blunting and A-tailing in agarose plugs. Modified BLESS adapters containing Illumina adapter sequences	Live cells (at least 10^7 cells)	AsiSI-induced DSBs resection mapping, RAG endonuclease specificity[5]
Digenome-seq	Direct	*In vitro* nuclease digestion of purified genomic DNA and detection of DSBs by whole-genome sequencing	Purified DNA	Cas9 and Cpf1 specificity[6,7]
ChIP-seq	Indirect	Capture of chromatin containing DSBs markers such as γH2A.X	Fixed cells (at least 10^7 cells)	Replication stress-induced DSBs in yeast[8], AsiSI-induced DSBs processing in mammalian cells[9], transcription-associated DSBs in neuronal cells[10]
GUIDE-seq	Indirect	*In vivo* DSB labeling by incorporation of dsDNA oligos through NHEJ-mediated repair	Transfected live cells	Cas9 and Cpf1 specificity[11,12]
IDLV capture	Indirect	*In vivo* DSB labeling by random incorporation of integration defective lentiviral vectors through NHEJ-mediated repair	Transduced live cells	Cas9 and TALENs specificity[13]
LAM-HTGTS	Indirect	*In vivo* induction of DSBs and sequencing of translocation products originated from NHEJ-mediated repair	Live cells treated to induce translocations	Cas9 specificity[14], transcription-associated DSBs in neuronal cells[15]

SUPPLEMENTARY REFERENCES

1. Crosetto, N. *et al.* Nucleotide-resolution DNA double-strand break mapping by next-generation sequencing. *Nat. Methods* **10,** 361–365 (2013).
2. Ran, F. A. *et al.* In vivo genome editing using Staphylococcus aureus Cas9. *Nature* **520,** 186–191 (2015).
3. Slaymaker, I. M. *et al.* Rationally engineered Cas9 nucleases with improved specificity. *Science* **351,** 84–88 (2016).

4. Lensing, S. V. *et al.* DSBCapture: in situ capture and sequencing of DNA breaks. *Nat. Methods* **13,** 855–857 (2016).
5. Canela, A. *et al.* DNA Breaks and End Resection Measured Genome-wide by End Sequencing. *Mol. Cell* **63,** 898–911 (2016).
6. Kim, D. *et al.* Genome-wide analysis reveals specificities of Cpf1 endonucleases in human cells. *Nat. Biotechnol.* **34,** 863–868 (2016).
7. Kim, D. *et al.* Digenome-seq: genome-wide profiling of CRISPR-Cas9 off-target effects in human cells. *Nat. Methods* **12,** 237–43 (2015).
8. Szilard, R. K. *et al.* Systematic identification of fragile sites via genome-wide location analysis of gamma-H2AX. *Nat. Struct. Mol. Biol.* **17,** 299–305 (2010).
9. Iacovoni, J. S. *et al.* High-resolution profiling of gammaH2AX around DNA double strand breaks in the mammalian genome. *EMBO J.* **29,** 1446–1457 (2010).
10. Madabhushi, R. *et al.* Activity-Induced DNA Breaks Govern the Expression of Neuronal Early-Response Genes. *Cell* **161,** 1592–1605 (2015).
11. Tsai, S. Q. *et al.* GUIDE-seq enables genome-wide profiling of off-target cleavage by CRISPR-Cas nucleases. *Nat. Biotechnol.* **33,** 187–197 (2015).
12. Kleinstiver, B. P. *et al.* Genome-wide specificities of CRISPR-Cas Cpf1 nucleases in human cells. *Nat. Biotechnol.* **34,** 869–874 (2016).
13. Wang, X. *et al.* Unbiased detection of off-target cleavage by CRISPR-Cas9 and TALENs using integrase-defective lentiviral vectors. *Nat. Biotechnol.* **33,** 175–178 (2015).
14. Frock, R. L. *et al.* Genome-wide detection of DNA double-stranded breaks induced by engineered nucleases. *Nat. Biotechnol.* **33,** 179–186 (2015).
15. Wei, P.-C. *et al.* Long Neural Genes Harbor Recurrent DNA Break Clusters in Neural Stem/Progenitor Cells. *Cell* **164,** 644–655 (2016).
